# Plasma Diacylglycerols Are Associated with Carotid Intima-Media Thickness Among Patients with Type 2 Diabetes: Findings from a Supercritical Fluid Chromatography/Mass Spectrometry-Based Semi-Targeted Lipidomic Analysis

**DOI:** 10.3390/ijms26146977

**Published:** 2025-07-20

**Authors:** Naohiro Taya, Naoto Katakami, Kazuo Omori, Shigero Hosoe, Hirotaka Watanabe, Mitsuyoshi Takahara, Kazuyuki Miyashita, Yutaka Konya, Sachiko Obara, Ayako Hidaka, Motonao Nakao, Masatomo Takahashi, Yoshihiro Izumi, Takeshi Bamba, Iichiro Shimomura

**Affiliations:** 1 Department of Metabolic Medicine, Graduate School of Medicine, Osaka University, 2-2, Yamadaoka, Suita 565-0871, Osaka, Japan; taya@endmet.med.osaka-u.ac.jp (N.T.); omori@endmet.med.osaka-u.ac.jp (K.O.); shosoe@endmet.med.osaka-u.ac.jp (S.H.); takahara@endmet.med.osaka-u.ac.jp (M.T.); kmiyas@endmet.med.osaka-u.ac.jp (K.M.); shimomura@endmet.med.osaka-u.ac.jp (I.S.); 2 Department of Diabetes Care Medicine, Graduate School of Medicine, Osaka University, 2-2, Yamadaoka, Suita 565-0871, Osaka, Japan; 3 Department of Laboratory Medicine, Graduate School of Medicine, Osaka University, 2-2, Yamadaoka, Suita 565-0871, Osaka, Japan; 4 Division of Metabolomics, Medical Research Center for High Depth Omics, Medical Institute of Bioregulation, Kyushu University, 3-1-1 Maidashi, Higashi-ku, Fukuoka 812-8582, Fukuoka, Japana.hidaka.uq@adm.fukuoka-u.ac.jp (A.H.); m-takahashi@bioreg.kyushu-u.ac.jp (M.T.); izumi@bioreg.kyushu-u.ac.jp (Y.I.); bamba@bioreg.kyushu-u.ac.jp (T.B.)

**Keywords:** diacylglycerol, intima-media thickness, lipidomics, type 2 diabetes, atherosclerosis

## Abstract

Abnormalities in plasma lipoproteins observed in patients with diabetes promote atherosclerosis. However, the association between various lipid species and classes and atherosclerosis remains unclear. Here, we aimed to identify the plasma lipid characteristics associated with atherosclerosis progression in patients with diabetes. We performed semi-targeted lipidomic analysis of fasting plasma samples using supercritical fluid chromatography coupled with mass spectrometry in two independent patient groups with type 2 diabetes (*n* = 223 and 31) and evaluated cross-sectional associations between plasma lipids and carotid intima-media thickness (CIMT). Ten plasma lipid species, including eight diacylglycerols (DGs), and total DG levels were significantly associated with CIMT in both groups. Patients of the former group were partly observed for 5 years, and we investigated associations between DGs and CIMT progression in these patients (*n* = 101). As a result, 22 DGs among the 26 identified DGs and total DG (*β* = 0.398, *p* < 0.001) were significantly associated with the annual change in CIMT. Furthermore, plasma DG levels improved the predictive ability for CIMT progression, with an adjusted R-squared increase of 0.105 [95% confidence interval: 0.010, 0.232] in the models. Plasma DGs are associated with CIMT progression in patients with type 2 diabetes. Measurement of total plasma DG levels may be beneficial in assessing the risk of atherosclerosis progression.

## 1. Introduction

The global prevalence of diabetes mellitus is high (10.5%), and the number of patients with diabetes is estimated to rise from 540 million to 780 million by 2045 [1]. Diabetes, caused by a relative or absolute deficiency of insulin, is a group of chronic metabolic disorders characterized by hyperglycemia. Patients with diabetes often develop various metabolic abnormalities, such as amino acid and lipid metabolic disorders, in addition to hyperglycemia. Regarding lipid metabolism, common abnormalities in patients with diabetes include elevated levels of VLDL, chylomicrons, and their remnants, along with decreased levels of HDL. These abnormalities of plasma lipoprotein are associated with the development of diabetic complications.

Cardiovascular disease (CVD), including myocardial infarction, peripheral artery disease and stroke, remains the main cause of death in patients with diabetes, and is associated with an increase in economic burden. Since CVD follows subclinical atherosclerosis as a consequence of its progression—and diabetes accelerates this process [2]—atherosclerosis, also known as diabetic macroangiopathy, is considered one of the major complications of diabetes.

Recent studies have reported that various lipid species and classes, composed of various fatty acids (FAs), are associated with atherosclerosis and CVD [3]. For example, certain ceramides and sphingolipids are associated with the development of CVD [3,4,5,6]. Some lipid species, including triacylglycerols (TGs), ceramides, and sphingolipids, are found to have an association with incident type 2 diabetes [7]. Basic research demonstrated that diacylglycerols (DGs) and ceramides were implicated in the pathogenesis of insulin resistance [8,9]. These findings suggest that specific lipid species and classes may be associated with the progression of diabetes and its complications, particularly diabetes-accelerated atherosclerosis. However, to date, no studies have comprehensively examined the association between various lipids and atherosclerosis in patients with diabetes.

Moreover, most previous lipidomic studies have evaluated lipids primarily based on the total number of carbon atoms and double bonds in their fatty acids (FAs), with few investigations exploring the detailed composition of individual FA constituents. We previously analyzed the detailed FA composition across various lipid classes using supercritical fluid chromatography coupled with tandem mass spectrometry (SFC/MS/MS) in patients with diabetes and demonstrated changes in the FA composition of plasma TGs following comprehensive diabetes risk management [10]. This lipidomic approach enables comprehensive and quantitative analysis, and its high repeatability and accuracy have been previously validated [11].

To identify lipid species associated with the development of diabetic macroangiopathy, we aimed to investigate the associations between various lipid molecules and carotid intima-media thickness (CIMT), a surrogate marker of CVD, in patients with diabetes. For this purpose, lipids containing a variety of FA constituents across multiple lipid classes were measured by semi-targeted lipidomic analysis using SFC/MS/MS.

## 2. Results

### 2.1. Associations Between Plasma Lipids and CIMT (Cross-Sectional Analyses)

First, we conducted a cross-sectional evaluation of the associations between plasma lipids, measured by semi-targeted lipidomic analysis, and CIMT in two independent patient groups with type 2 diabetes (group 1 and 2). The clinical characteristics of the participants are shown in Table 1. Almost half of the participants were men (57.4% in group 1 and 41.9% in group 2). The mean age (± standard deviation) was 60.6 ± 11.4 years in group 1 and 63.9 ± 10.5 years in group 2. Participants in both groups had relatively high BMIs of 27.7 ± 5.9 and 26.7 ± 5.1 kg/m^2^, respectively. Their glycemic control was relatively poor, with glycated hemoglobin (HbA1c) levels of 9.1 ± 1.8% (75.5 ± 19.7 mmol/mol) and 9.1 ± 2.1% (76.4 ± 22.6 mmol/mol). CIMT values were 1.9 ± 0.8 and 2.0 ± 1.0 mm, respectively.

In the plasma lipidomic analysis of group 1 conducted using SFC/MS/MS, 349 lipids belonging to 16 lipid classes were identified (Table S1). The associations between these lipid species and classes and CIMT are shown in Table S1. As listed in Table 2, 22 lipid species, including 16 DGs, and total DG were significantly associated with CIMT (*p* < 0.05). Since 16 of these 22 lipid species were also detected in the plasma of group 2 patients, we further examined their association with CIMT in group 2. As a result, eight DGs (DG 16:0_16:0, DG 16:0_18:1, DG 16:0_18:2, DG 16:0_18:3, DG 16:1_18:2, DG 18:1_18:1, DG 18:1_18:2, DG 18:1_18:3), phosphatidylglycerol (PG) 16:0_18:1, and TG 16:0_16:0_18:0 were significantly associated with CIMT (*p* < 0.05) (Table 2). Total DG levels also showed a significant association with CIMT (*p* < 0.05) in group 2.

### 2.2. Associations Between Plasma DGs and the Progression of CIMT (Longitudinal Analyses)

Based on the above cross-sectional analyses, we focused on DG from the lipid classes identified in our lipidomic analyses due to the following two points. First, many species of DGs showed cross-sectional associations with CIMT. Second, total DG levels were also associated with CIMT. In contrast, only a single lipid species from each of the phosphatidylglycerol (PG) and triglyceride (TG) classes was associated with CIMT. To further investigate whether plasma DGs were associated with CIMT progression, we conducted a longitudinal analysis within a prospective observational study over a 5-year period. Patients with type 2 diabetes without coronary artery disease (CAD) who underwent carotid ultrasonography at baseline and after 3 or 5 years were included in this analysis (group 3).

The baseline clinical characteristics of the participants in this analysis are shown in Table 3. The participants had a mean age of 60.8 ± 11.5 years, 46.6% were men, HbA1c was 9.0 ± 1.7% (75.4 ± 18.7 mmol/mol), and BMI was 27.7 ± 6.1 kg/m^2^. A total of 80.6% of participants underwent carotid ultrasonography three times, while the remainder underwent it twice. The annual change in CIMT was 0.015 ± 0.131 mm/year.

Among the 26 identified DGs, 22 were significantly associated with the annual change in CIMT (*p* < 0.05), independent of major conventional CVD risk factors, such as age, sex, BMI, diabetes duration, hypertension, HbA1c, smoking status, statin use, and baseline CIMT (model 1 in Table 4). Total DG was also significantly associated with the annual change in CIMT (*β* = 0.398, *p* < 0.001) (model 1 in Table 4). Similar results were observed after adjustment for HDL cholesterol, LDL cholesterol, and log-transformed triglyceride levels (model 2 in Table 4).

Finally, we investigated whether adding total DG values to the standard atherosclerosis prediction model, which includes the Suita score (an established Japanese risk score for coronary heart disease) [12], HbA1c levels, and baseline CIMT, could improve the predictive ability for annual CIMT change. The adjusted R-squared values for the models before and after the inclusion of total DG values were 0.101 and 0.206, respectively (Table 5), showing a statistically significant improvement (increase of 0.105; 95% confidence interval (CI) 0.010, 0.232). In contrast, adding serum triglyceride levels measured by standard techniques to the standard model did not result in a statistically significant improvement (the adjusted R-squared increase was 0.030; 95% CI −0.009, 0.122).

## 3. Discussion

Based on two independent cross-sectional studies, we found that high plasma DG levels are associated with CIMT thickening in patients with type 2 diabetes. In addition, a prospective observational study revealed that high plasma DG levels at the beginning of observation were a risk factor for subsequent CIMT thickening. We further confirmed that assessing total plasma DG levels, in addition to classical risk factors for atherosclerosis, significantly improved the predictive ability for CIMT progression. These results suggest that high plasma DG levels may contribute to atherosclerosis progression in patients with type 2 diabetes and that measuring total plasma DG levels could be useful in assessing future atherosclerosis risk. This study is the first to measure various plasma DGs, particularly those composed of various combinations of constituent FAs, in patients with type 2 diabetes and to establish their association with atherosclerosis.

The results of this study are generally consistent with those of previous clinical studies. For example, Meikle et al. performed a cross-sectional lipidomic study, showing that plasma levels of total DG are higher in patients with stable CAD than in healthy controls [13]. Another cross-sectional study showed that blood LDL from patients with acute coronary syndrome contained more DG 18:0_22:6 than that from patients with stable CAD [14]. Similarly, Toledo E et al. analyzed plasma lipidomic profiles in a randomized intervention trial and reported associations between plasma DG 34:1, DG 34:2, DG 36:0, and DG 36:1 levels and the risk of CVD in patients at high risk for CVD [15]. A lipidomic study using carotid ultrasonography revealed that plasma levels of DG 18:1_20:4 were cross-sectionally associated with the echogenicity of the intima-media complex, but not CIMT, in a population-based cohort in Sweden [16]. Another ultrasonographic study examined the association between various lipid species at baseline and risk of carotid artery plaque in multicenter HIV cohorts without diabetes and found that 120 lipid species including DGs were associated with the incident carotid artery plaque [17]. However, our study differs significantly from these previous reports in that we exclusively focused on patients with type 2 diabetes, measured a variety of plasma DGs consisting of combinations of various FA chains rather than specific DGs, and demonstrated associations between these diverse plasma DGs and atherosclerosis in both cross-sectional and longitudinal analyses.

Interestingly, matrix-assisted laser desorption/ionization mass spectrometry imaging of human carotid atherosclerotic plaques has revealed that DGs accumulate in areas containing the thrombus-associated protein fibrin [18], suggesting that DGs are involved in coagulation during the progression of atherosclerosis. However, whether the deposited DGs originate from plasma or are synthesized in cells, such as endothelial cells (ECs) and platelets, is unclear. In these cells, DGs can be either synthesized during de novo lipid biosynthesis or generated from other intracellular lipid species [19].

Several mechanisms by which DGs promote atherosclerosis have been elucidated. For example, DGs activate protein kinase C (PKC) in various cell types, including ECs, vascular smooth muscle cells (VSMCs), macrophages [20], and platelets [21]. In ECs, PKC contributes to endothelial dysfunction by regulating nitric oxide production [22] and inflammatory responses [23]. In macrophages, PKC regulates cholesterol efflux [24], promotes LDL uptake, and oxidizes LDL, leading to foam cell formation [25]. Moreover, PKC plays a role in the proliferation and apoptosis of VSMCs [20] and platelet activation [21]. Thus, in these vascular cells, DGs may promote atherosclerosis via PKC activation, although whether plasma DGs are directly taken up by these cells is unknown.

A recent study has demonstrated that DG exposure accelerates the secretion of proinflammatory cytokines from human monocytes. This activation of monocytes by DGs enhances their adhesion to ECs and promotes transendothelial migration [26]. Thus, plasma DGs as well as DGs within vascular cells may promote atherosclerosis.

Non-alcoholic fatty liver disease (NAFLD), which is strongly associated with type 2 diabetes mellitus, may coexist with high plasma DG levels and atherosclerosis. In patients with NAFLD, DG accumulation in the liver promotes insulin resistance via the activation of PKCε [9]. Increased insulin resistance in the liver exacerbates atherosclerosis by increasing hepatic de novo lipogenesis, leading to the overproduction of triglyceride-rich lipoproteins, including VLDL, and altering glucose metabolism [27,28]. The dysregulation of lipoprotein and glucose metabolism also contributes to ectopic fat accumulation, which is closely associated with atherosclerotic diseases [29]. Recent studies have demonstrated that NAFLD leads to an increase in hepatic secretion of DGs, resulting in increased plasma DG levels [30]. Consequently, increased DGs in the liver may cause high plasma DG levels and further drive atherosclerosis development.

The present study has several limitations. First, detailed patient information regarding lifestyle-related factors, such as diet, physical activity, and alcohol consumption, was not available, despite the significant influence these factors can have on plasma lipid levels and diabetic macrovascular complications. Second, as this was a small-sample study conducted at a single center, the reliability of the results may be limited. Third, CIMT increases over time with the progression of atherosclerosis [31], but non-atherosclerotic compensatory enlargement of the carotid wall is also reflected in CIMT thickening. Thus, CIMT was not completely correlated with the risk for CVD, although both CIMT itself and its change over time are prognostic factors for CVD [32].

Nevertheless, the study yielded meaningful findings, suggesting that plasma total DG could serve as a potential biomarker for atherosclerosis. Future large-scale studies with cardiovascular events as outcomes are needed to further evaluate the potential of plasma DG as a biomarker for CVD. In addition, the development of a simpler method for measuring total DG is also required, since the semi-targeted lipidomic analysis used in this study is not suitable for clinical application due to its cost and complexity. Furthermore, our results support the hypothesis that plasma DG can promote atherosclerosis development. As DG is an intermediate in TG hydrolysis, it may be abundant in remnant lipoproteins, which are highly atherogenic. Therefore, the atherogenic properties of remnant lipoproteins may be partially attributed to DG. Understanding the mechanism underlying DG’s atherogenicity could eventually lead to the identification of novel therapeutic targets.

## 4. Materials and Methods

### 4.1. Study Design and Participants

This study used data from two previous studies, both of which conducted plasma lipidomic analysis using SFC/MS/MS. The first study investigated the association between plasma metabolites and CAD [33]. This study included patients with type 2 diabetes aged 20 to 75 years. Patients who should not receive strict glycemic management, such as those who frequently underwent severe hypoglycemia and those with unstable diabetic retinopathy, those whose serum creatinine level was over 2.0 mg/dL, those with severe infection or trauma, and perioperative patients were excluded. Patients who met eligibility criteria were consecutively recruited at Osaka University Hospital. From the 240 patients originally enrolled between June 2014 and October 2016 (study 1), those who underwent carotid ultrasonography at baseline were included in the present analysis (*n* = 223, group 1). The second study was a prospective observational study that identified short-term changes in the metabolome of inpatients receiving comprehensive diabetes treatment (study 2) [34]. This study included the patients with type 2 diabetes who were admitted to Osaka University Hospital to improve glycemic management and aged between 20 and 75 years, and the exclusion criteria were the same as those of study 1. Thirty-one inpatients were enrolled between November 2017 and February 2019, and all were included in this analysis (group 2). Moreover, participants from study 1 included 40 patients with a history of CAD and 200 patients without CAD. The latter group was prospectively followed for 5 years. For the longitudinal analysis, patients without CAD who underwent carotid ultrasonography at baseline and after 3 or 5 years were selected (*n* = 103, group 3).

The protocols of the original studies were approved by the Ethics Committee of Osaka University Hospital (approval numbers 13454 and 16374). The studies were conducted in accordance with the principles of the Declaration of Helsinki and current legal regulations in Japan. Written informed consent was obtained from all participants following a full explanation of the study.

### 4.2. Study Protocol

Clinical data collected from participants included age, sex, diabetes duration, smoking status (never or ever smoked), comorbidities, and medications. Height and weight were measured, and blood samples were collected after overnight fasting. Total cholesterol, HDL cholesterol, LDL cholesterol, and triglyceride levels were determined using standard techniques. The estimated glomerular filtration rate was calculated according to the guidelines of the Japanese Society of Nephrology [35].

For lipidomic analysis, portions of the blood samples were cooled at 4 °C immediately after collection. The samples were then centrifuged (3000× *g*, 10 min), and the plasma was stored at −80 °C within 4 h of collection.

### 4.3. Measurement of CIMT

Ultrasonographic scans of the carotid artery were conducted using a high-resolution B-mode ultrasound scanner equipped with a high-frequency (>7.5 MHz) linear transducer with a detection limit of <0.1 mm. All scans were performed by experienced laboratory physicians using a standardized measurement method in accordance with the guidelines of the Japan Society of Ultrasonics in Medicine [36]. The common carotid artery, carotid bulb, and internal carotid artery were scanned bilaterally, and the thickest points of the intima-media thickness in each section were measured separately. The highest measurement was defined as the CIMT, representing the value for each individual. We previously checked the reproducibility analysis of replicate measurements in the randomly selected 20 subjects and confirmed that the absolute mean difference and coefficients of variation for the measurements of CIMT were 0.01 ± 0.01 and 0.7%, respectively.

### 4.4. Lipidomic Measurement

For lipid extraction, plasma samples were prepared following the Bligh and Dyer method [37], with minor modifications, as previously described [10]. Lipids were extracted from 30 μL of plasma with 470 μL of methanol containing the internal standards described in Tables S2 and S3. The samples were then vigorously mixed for 1 min and centrifuged at 16,000× *g* for 5 min at 4 °C, after which the supernatant (400 μL) was collected. Subsequently, 400 μL of chloroform and 224 μL of water were added, and the aqueous and organic layers were separated by further mixing and centrifugation at 16,000× *g* for 5 min at 4 °C. The organic layer (300 μL), obtained from the bottom phase, was dried under a nitrogen stream and stored at −80 °C until analysis. Prior to analysis, the dried sample was reconstituted in methanol/chloroform (1/1, *v*/*v*, 200 μL).

Lipid analyses in the two original studies were independently conducted using an SFC system (Nexera UC system; Shimadzu Corporation, Kyoto, Japan) coupled to a triple quadrupole mass spectrometer (LCMS-8060; Shimadzu) operating in multiple reaction monitoring (MRM) mode, as previously described [10,38,39]. An ACQUITY UPC2 Torus diethylamine (DEA) column (3.0 mm i.d. × 100 mm, 1.7 μM particle size; Waters Corporation, Milford, CT, USA) and an ACQUITY UPC2 HSS C18 SB column (3.0 mm i.d. × 100 mm, 1.8 μM particle size, Waters) were used for separation, depending on the lipid class. Data processing was performed using LabSolutions software (version 5.99 SP2; Shimadzu). Details of the analytical conditions for lipid analyses are described in the Appendices A.1 and A.2.

A reference sample was prepared by mixing equal amounts (10 µL each) of plasma extracts from the participants in each study. A total of 20 FA constituents of TGs and 35 FA constituents of other lipid classes were selected using SFC/MS/MS analysis of the hydrolyzed reference sample in study 1 [38]. To determine the target lipids in each study, the reference sample was analyzed using an in-house MRM library for lipids composed of the selected FA constituents [11]. Targeted quantitative analysis was then performed on the determined lipids, belonging to 16 lipid classes, including cholesterol, free fatty acid (FFA), DG, TG, cholesterol ester (CE), phosphatidylcholine (PC), phosphatidylethanolamine (PE), phosphatidylinositol (PI), PG, phosphatidylserine (PS), lysophosphatidylcholine (LPC), lysophosphatidylethanolamine (LPE), lysophosphatidylinositol (LPI), ceramide (Cer), hexosylceramide (HexCer), and sphingomyelin (SM), using the selected MRM method [32,33].

### 4.5. Statistical Analysis

Clinical data are expressed as means and standard deviations for normally distributed data, or as medians and interquartile ranges for log-normally distributed data. Categorical variables are expressed as counts and percentages. Statistical significance was set at *p* < 0.05.

The plasma level of each lipid class was calculated as the sum of the lipid species within that class. All lipid data, including plasma levels of lipid species and classes, were log-transformed for statistical analysis.

First, cross-sectional analyses were separately performed for groups 1 and 2. To identify lipids associated with CIMT, we evaluated the association between all lipid species and classes and CIMT using a linear regression model among the patients in group 1. This model was adjusted for age, sex, BMI, diabetes duration, HbA1c, presence of hypertension, smoking status (never or ever smoked), and statin use. We then validated whether the lipids found to be associated with CIMT in group 1 were similarly associated with CIMT in group 2.

Next, we conducted a longitudinal study to assess whether the lipids on which we focus based on the cross-sectional analyses were associated with CIMT progression. The annual change in CIMT was calculated from values at baseline and after 3 and 5 years, using the least-squares method. The association between baseline lipids and the annual change in CIMT among patients in group 3 was evaluated using multivariate linear regression analyses with two models. Model 1 was adjusted for age, sex, baseline CIMT, BMI, diabetes duration, hypertension, HbA1c, smoking status (never or ever smoked), and statin use. Model 2 was adjusted for HDL cholesterol, LDL cholesterol, log-transformed triglycerides and the variables included in model 1.

Finally, we examined whether adding lipid-related parameters to the standard atherosclerosis prediction model, which was created using the Suita score [12], HbA1c, and baseline CIMT, improved the model’s predictive ability for CIMT progression. To assess model fit, the adjusted R-squared values before and after adding lipid-related parameters were calculated and compared. The 95% CI of the difference in adjusted R-squared between the models was estimated using the bootstrap method with the R package boot (R Development Core Team, Vienna, Austria).

Statistical analyses were performed using SPSS Statistics (version 25; IBM Corporation, Armonk, NY, USA) and R statistical software (version 4.3.1; R Development Core Team).

## 5. Conclusions

Our lipidomic study demonstrated that high plasma DG levels were associated with CIMT thickening in patients with type 2 diabetes. These findings may contribute to elucidating the pathophysiology of diabetic macroangiopathy and identifying novel therapeutic targets, although further studies are warranted. Additionally, we confirmed that incorporating total plasma DG levels into a standard atherosclerosis prediction model improved its ability to predict CIMT progression. This suggests that assessing plasma DG levels may be useful for risk stratification to identify patients at high risk of atherosclerotic complications.

## Figures and Tables

**Table 1:** Clinical characteristics of the study participants in the cross-sectional analysis.

	Group 1 (*n* = 223)	Group 2 (*n* = 31)
Sex: male	128 (57.4)	13 (41.9)
Age (years)	60.6 ± 11.4	63.9 ± 10.5
Diabetes duration (year)	13.0 ± 9.7 *	16.8 ± 11.1
Ever smoker	118 (52.9) *	15 (48.4)
BMI (kg/m^2)^	27.7 ± 5.9	26.7 ± 5.1
Hypertension	147 (65.9)	22 (71.0)
Dyslipidemia	150 (67.3)	26 (83.9)
CAD	35 (15.7)	7 (22.6)
Statin user	98 (43.9)	22 (71.0)
FPG (mmol/L)	8.54 ± 2.90 †	8.07 ± 2.56
HbA1c (mmol/mol)	75.5 ± 19.7 *	76.4 ± 22.6
HbA1c (%)	9.1 ± 1.8 *	9.1 ± 2.1
AST (U/L)	29.8 ± 23.1	27.9 ± 17.1
ALT (U/L)	32.0 ± 26.4	30.6 ± 27.4
γ-GTP (U/L)	54.5 ± 74.1 *	39.1 ± 30.4
eGFR (ml min^−1^ 1.73 m^−2^)	72.7 ± 24.0	70.8 ± 21.5
Uric acid (mmol/L)	0.36 ± 0.09	0.34 ± 0.07
Total cholesterol (mmol/L)	5.11 ± 1.30	5.20 ± 1.58
LDL cholesterol (mmol/L)	2.97 ± 0.99	3.11 ± 1.26
HDL cholesterol (mmol/L)	1.25 ± 0.37	1.37 ± 0.31
Triglycerides (mmol/L)	1.56 (1.07–2.53)	1.41 (0.89–2.66)
u-Alb/Cr (mg/mmol)	1.38 (0.55–7.40) ‡	1.41 (0.38–4.52)
CIMT (mm)	1.9 ± 0.8	2.0 ± 1.0

Data are presented as means ± standard deviations for normally distributed or as medians (interquartile ranges) for log-normally distributed. Categorical data are presented as counts (percentages). * n = 222, † n = 220, ‡ n = 218. CAD, coronary artery disease; FPG, fasting plasma glucose; AST, aspartate aminotransferase; ALT, alanine aminotransferase; γ-GTP, γ-glutamyl transpeptidase; eGFR, estimated glomerular filtration rate; u-Alb/Cr, urine albumin/creatinine ratio; CIMT, carotid intima-media thickness.

**Table 2:** Associations between plasma lipid levels and carotid intima-media thickness (CIMT) in the cross-sectional analysis.

	Group 1 (*n* = 220)		Group 2 (*n* = 31)	
	*β*	*p*-Value	*β*	*p*-Value
DG 16:0_16:0	0.155	0.029	0.519	0.006
DG 16:0_18:0	0.188	0.008	NA	-
DG 16:0_18:1	0.178	0.014	0.595	0.001
DG 16:0_18:2	0.180	0.011	0.577	0.002
DG 16:0_18:3	0.160	0.017	0.531	0.004
DG 16:0_20:4	0.145	0.041	NA	-
DG 16:1_18:2	0.130	0.048	0.451	0.013
DG 17:1_18:2	0.175	0.008	NA	-
DG 18:0_18:1	0.153	0.034	0.379	0.056
DG 18:1_18:1	0.146	0.040	0.493	0.008
DG 18:1_18:2	0.151	0.028	0.429	0.032
DG 18:1_18:3	0.146	0.029	0.392	0.048
DG 18:1_20:3	0.140	0.047	NA	-
DG 18:1_20:4	0.143	0.036	0.284	0.226
DG 18:2_18:2	0.136	0.041	0.286	0.170
DG 18:2_18:3	0.148	0.021	0.321	0.101
total DG	0.167	0.017	0.512	0.007
PC 18:2_20:1	0.144	0.027	−0.016	0.927
PE 16:0_18:0	0.157	0.014	NA	-
PE 17:0_18:2	0.128	0.045	NA	-
PE 18:1_18:1	0.134	0.038	0.278	0.126
PG 16:0_18:1	0.128	0.048	0.509	0.006
TG 16:0_16:0_18:0	−0.122	0.049	0.486	0.007

Linear regression analysis was performed to evaluate the associations between lipids and CIMT among patients in groups 1 and 2. The analysis in group 1 was adjusted for age, sex, BMI, diabetes duration, hypertension, HbA1c, smoking status (never or ever), and statin use. The analysis in group 2 was adjusted for age, sex, and statin use. Only the lipids associated with CIMT among the patients in group 1 are listed in this table. *β*, standard partial regression coefficient; DG, diacylglycerol; PC, phosphatidylcholine; PE, phosphatidylethanolamine; PG, phosphatidylglycerol; TG, triacylglycerol; NA, not assessed, since the lipid was not identified in the lipidomic analysis of plasma from patients in group 2.

**Table 3:** Clinical characteristics of the study participants in the longitudinal analysis.

	Group 3 (*n* = 103)
Sex: male	48 (46.6)
Age (years)	60.8 ± 11.5
Diabetes duration (year)	13.3 ± 9.7
Ever smoker	46 (45.1) *
BMI (kg/m^2^)	27.7 ± 6.1
Hypertension	66 (64.1)
Dyslipidemia	70 (68.0)
Statin user	42 (40.8)
FPG (mmol/L)	8.41 ± 3.37
HbA1c (mmol/mol)	75.4 ± 18.7
HbA1c (%)	9.0 ± 1.7
AST (U/L)	32.0 ± 21.5
ALT (U/L)	33.9 ± 25.9
γ-GTP (U/L)	57.7 ± 87.0
eGFR (ml min^−1^ 1.73 m^−2^)	77.0 ± 23.1
Uric acid (mmol/L)	0.35 ± 0.08
Total cholesterol (mmol/L)	5.19 ± 1.24
LDL cholesterol (mmol/L)	3.09 ± 0.98
HDL cholesterol (mmol/L)	1.26 ± 0.33
Triglycerides (mmol/L)	1.54 (1.03–2.52)
u-Alb/Cr (mg/mmol)	1.26 (0.56–3.64) †
Suita score	49.1 ± 10.2 ‡
Number of carotid ultrasonographies (twice/three times)	20 (19.4)/83 (80.6)
Baseline CIMT (mm)	1.8 ± 0.8
Annual change in CIMT (mm/year)	0.015 ± 0.131

Data are presented as means ± standard deviations for normally distributed or as medians (interquartile ranges) for log-normally distributed. Categorical data are presented as counts (percentages). * n = 102, † n = 100, ‡ n = 99. FPG, fasting plasma glucose; AST, aspartate aminotransferase; ALT, alanine aminotransferase; γ-GTP, γ-glutamyl transpeptidase; eGFR, estimated glomerular filtration rate; u-Alb/Cr, urine albumin/creatinine ratio; CIMT, carotid intima-media thickness.

**Table 4:** Associations between plasma diacylglycerols (DGs) and the annual change in CIMT.

	Model 1 (*n* = 102)		Model 2 (*n* = 102)	
	*β*	*p*-Value	*β*	*p*-Value
DG 12:0_18:1	0.131	0.227	0.013	0.919
DG 12:0_18:2	0.156	0.150	0.046	0.722
DG 14:0_16:0	0.119	0.280	−0.030	0.829
DG 14:0_18:1	0.228	0.038	0.134	0.397
DG 14:0_18:2	0.252	0.019	0.177	0.254
DG 16:0_16:0	0.184	0.119	0.005	0.975
DG 16:0_16:1	0.246	0.027	0.154	0.326
DG 16:0_18:0	0.284	0.017	0.193	0.306
DG 16:0_18:1	0.318	0.008	0.284	0.153
DG 16:0_18:2	0.381	0.001	0.425	0.019
DG 16:0_18:3	0.286	0.009	0.231	0.161
DG 16:0_20:4	0.296	0.011	0.230	0.164
DG 16:0_20:5	0.215	0.037	0.144	0.223
DG 16:1_18:1	0.365	0.001	0.492	0.004
DG 16:1_18:2	0.377	<0.001	0.523	0.002
DG 17:1_18:2	0.402	<0.001	0.509	0.001
DG 18:0_18:1	0.409	<0.001	0.580	0.002
DG 18:1_18:1	0.370	0.001	0.455	0.012
DG 18:1_18:2	0.401	<0.001	0.486	0.002
DG 18:1_18:3	0.373	<0.001	0.440	0.004
DG 18:1_20:3	0.376	0.001	0.416	0.004
DG 18:1_20:4	0.351	0.001	0.348	0.016
DG 18:1_20:5	0.249	0.012	0.192	0.083
DG 18:2_18:2	0.373	<0.001	0.382	0.006
DG 18:2_18:3	0.363	<0.001	0.389	0.005
DG 18:2_20:4	0.340	0.001	0.306	0.017
total DG	0.398	<0.001	0.586	0.002

Linear regression analysis was performed to evaluate the associations between DGs and the annual change in CIMT among the patients in group 3. Model 1 was adjusted for age, sex, BMI, diabetes duration, hypertension, HbA1c, smoking status (never or ever), and statin use. Model 2 was adjusted for HDL cholesterol, LDL cholesterol, log-transformed triglycerides, and covariates in model 1. *β*, standard partial regression coefficient; DG, diacylglycerol.

**Table 5:** Comparison of the predictive ability for the annual change in CIMT before and after adding lipid-related parameters.

	Added Lipid-Related Parameters	*R* ^2^ *_adj_*	∆*R*^2^*_adj_* (95% CI)
Model 1	None	0.101	Reference
Model 2	Log-transformed triglycerides	0.131	0.030 (−0.009, 0.122)
Model 3	Total DG	0.206	0.105 (0.010, 0.232)

Adjusted R-squared values for the linear regression models to predict the annual change in CIMT was calculated (*n* = 99). Each prediction model includes the Suita score, HbA1c, baseline CIMT, and the added lipid-related parameter in the table. The 95% CI of the difference in adjusted R-squared was estimated using the bootstrap method. *R*^2^*_adj_*, adjusted R-squared value; CI, confidence interval; DG, diacylglycerol.

## Data Availability

The data presented in this study are not publicly available due to the protocols of the original studies and patient privacy considerations but are available from the corresponding author on reasonable request.

## Supplementary Materials

The following supporting information can be downloaded at https://www.mdpi.com/article/10.3390/ijms26146977/s1.

## Abbreviations

The following abbreviations are used in this manuscript:

CIMT	carotid intima-media thickness
DG	diacylglycerol
FA	fatty acid
CVD	cardiovascular disease
HbA1c	glycated hemoglobin
SFC/MS/MS	supercritical fluid chromatography coupled with tandem mass spectrometry
PG	phosphatidylglycerol
TG	triacylglycerol
CAD	coronary artery disease
CI	confidence interval
EC	endothelial cell
PKC	protein kinase C
VSMC	vascular smooth muscle cell
NAFLD	non-alcoholic fatty liver disease
MRM	multiple reaction monitoring
DEA	diethylamine
FFA	free fatty acid
CE	cholesterol ester
PC	phosphatidylcholine
PE	phosphatidylethanolamine
PI	phosphatidylinositol
PS	phosphatidylserine
LPC	lysophosphatidylcholine
LPE	lysophosphatidylethanolamine
LPI	lysophosphatidylinositol
Cer	ceramide
HexCer	hexosylceramide
SM	sphingomyelin

## Appendix A

### Appendix A.1. DEA-SFC/MS/MS Analysis for Determination of Monoacylglycerols, DGs, PCs, PEs, PIs, PGs, PSs, Phosphatidic Acids, LPCs, LPEs, LPIs, Cers, HexCers, and SMs

The DEA-SFC conditions were as follows: injection volume, 2 μL; column temperature, 50 °C; mobile phase A, supercritical carbon dioxide; mobile phase B (modifier) and make-up pump solvent; methanol/water (95/5, *v*/*v*) with 0.1% (*w*/*v*) ammonium acetate; flow rate of mobile phase, 1.0 mL/min; flow rate of make-up pump, 0.05 mL/min for study 1 and 0.1 mL/min for study 2; and back pressure regulator, 10 MPa. The gradient conditions were as follows: 1% B, 0−1 min; 1−75% B, 1−24 min; 75% B, 24−26 min; and 1% B, 26.1−30 min. The triple quadrupole mass spectrometer (TQMS) analysis conditions were as follows: polarity, positive and negative ionization; electrospray voltage, 4.0 kV in the positive ion mode and −3.5 kV in the negative ion mode; nebulizer gas flow rate, 3.0 L/min; drying gas flow rate, 10.0 L/min; desolvation line temperature, 250 °C; heat block temperature, 400 °C; and detector voltage, 2.16 kV. The MRM parameters per one time-period were as follows: limit on the number of MRM transitions, 150; dwell time, 2 milliseconds; and pause time, 2 milliseconds.

### Appendix A.2. C18-SFC/MS/MS Analysis for Determination of FFAs, TGs, CEs, and Cholesterol

The C18-SFC gradient conditions were as follows: 0−50% B, 0−25 min; 50% B, 25−28 min; and 0% B, 28.1−30 min. The other C18-SFC and TQMS parameters were the same as those used in the DEA-SFC/MS/MS analysis for study 1 and 2.
