# Supplementary material for: Plasma Diacylglycerols Are Associated with Carotid Intima-Media Thickness Among Patients with Type 2 Diabetes: Findings from a Supercritical Fluid Chromatography/Mass Spectrometry-Based Semi-Targeted Lipidomic Analysis
Source: Int J Mol Sci. 2025 Jul 20;26(14):6977. doi: 10.3390/ijms26146977 (PMC12295859; doi:10.3390/ijms26146977)
Supplement: Supplementary file 1 [file ijms-26-06977-s001.zip › ijms-3734859-supplementary.pdf]

Supplementary Tables

Plasma Diacylglycerols Are Associated with Carotid Intima-Media Thickness Among Patients with Type 2 Diabetes: Findings from a Supercritical Fluid Chromatography/Mass Spectrometry-Based Semi-Targeted Lipidomic Analysis

Naohiro Taya [1,2], Naoto Katakami [1,*], Kazuo Omori [1], Shigero Hosoe [1], Hirotaka Watanabe [1], Mitsuyoshi Takahara [1,3], Kazuyuki Miyashita [1], Yutaka Konya [4], Sachiko Obara [4], Ayako Hidaka [4], Motonao Nakao [4], Masatomo Takahashi [4], Yoshihiro Izumi [4], Takeshi Bamba [4] and Iichiro Shimomura [1]

[1] Department of Metabolic Medicine, Graduate School of Medicine, Osaka University, 2-2, Yamadaoka, Suita 565-0871, Osaka, Japan; taya@endmet.med.osaka-u.ac.jp (N.T.); omori@endmet.med.osaka-u.ac.jp (K.O.); shosoe@endmet.med.osaka-u.ac.jp (S.H.); takahara@endmet.med.osaka-u.ac.jp (M.T.); kmiyas@endmet.med.osaka-u.ac.jp (K.M.); shimomura@endmet.med.osaka-u.ac.jp (I.S.)

[2] Department of Diabetes Care Medicine, Graduate School of Medicine, Osaka University, 2-2, Yamadaoka, Suita 565-0871, Osaka, Japan

[3] Department of Laboratory Medicine, Graduate School of Medicine, Osaka University, 2-2, Yamadaoka, Suita 565-0871, Osaka, Japan

[4] Division of Metabolomics, Medical Research Center for High Depth Omics, Medical Institute of Bioregulation, Kyushu University, 3-1-1 Maidashi, Higashi-ku, Fukuoka 812-8582, Fukuoka, Japan; a.hidaka.uq@adm.fukuoka-u.ac.jp (A.H.); m-takahashi@bioreg.kyushu-u.ac.jp (M.T.); izumi@bioreg.kyushu-u.ac.jp (Y.I.); bamba@bioreg.kyushu-u.ac.jp (T.B.)

* Correspondence: katakami@endmet.med.osaka-u.ac.jp; Tel.: +81-6-6879-3743

Table S1 Associations between all identified lipids and carotid intima-media thickness among patients in group 1

	β	p-value
FFA 12:0	-0.021	0.740
FFA 14:0	0.013	0.835
FFA 14:1	0.029	0.647
FFA 16:0	0.018	0.768
FFA 16:1	-0.009	0.895
FFA 17:0	0.045	0.470
FFA 17:1	0.007	0.919
FFA 18:0	0.040	0.520
FFA 18:1	-0.002	0.971
FFA 18:2	0.001	0.985
FFA 18:3	0.006	0.924
FFA 19:0	-0.037	0.556
FFA 19:1	0.047	0.461
FFA 20:0	0.000	0.994
FFA 20:1	0.060	0.351
FFA 20:2	0.006	0.921
FFA 20:3	0.007	0.915
FFA 20:4	0.023	0.709
FFA 20:5	0.040	0.534
FFA 22:1	0.093	0.149
FFA 22:2	-0.051	0.410
FFA 22:3	-0.022	0.715
FFA 22:4	-0.001	0.983
FFA 22:5	0.005	0.940
FFA 22:6	-0.030	0.627
FFA 23:0	0.059	0.345
FFA 24:0	0.025	0.698
FFA 24:1	0.074	0.248
total FFA	0.013	0.830
DG 12:0_18:1	0.050	0.444
DG 12:0_18:2	0.094	0.149
DG 14:0_16:0	0.077	0.244
DG 14:0_18:1	0.099	0.138
DG 14:0_18:2	0.111	0.092

DG 16:0_16:0	0.155	0.029
DG 16:0_16:1	0.119	0.080
DG 16:0_18:0	0.188	0.008
DG 16:0_18:1	0.178	0.014
DG 16:0_18:2	0.180	0.011
DG 16:0_18:3	0.160	0.017
DG 16:0_20:4	0.145	0.041
DG 16:0_20:5	0.084	0.183
DG 16:1_18:1	0.128	0.057
DG 16:1_18:2	0.130	0.048
DG 17:1_18:2	0.175	0.008
DG 18:0_18:1	0.153	0.034
DG 18:1_18:1	0.146	0.040
DG 18:1_18:2	0.151	0.028
DG 18:1_18:3	0.146	0.029
DG 18:1_20:3	0.140	0.047
DG 18:1_20:4	0.143	0.036
DG 18:1_20:5	0.094	0.132
DG 18:2_18:2	0.136	0.041
DG 18:2_18:3	0.148	0.021
DG 18:2_20:4	0.118	0.077
total DG	0.167	0.017
TG 14:0_14:0_14:0	0.014	0.827
TG 14:0_14:0_16:0	0.028	0.661
TG 14:0_14:0_16:1	0.019	0.762
TG 14:0_14:0_18:0	0.028	0.663
TG 14:0_14:0_18:1	0.044	0.499
TG 14:0_14:0_18:2	0.059	0.358
TG 14:0_16:0_16:0	0.026	0.686
TG 14:0_16:0_16:1	0.043	0.504
TG 14:0_16:0_18:0	-0.018	0.800
TG 14:0_16:0_18:1	0.042	0.533
TG 14:0_16:0_18:2	0.071	0.272
TG 14:0_16:0_20:4	0.085	0.184
TG 14:0_16:0_20:5	0.070	0.272
TG 14:0_16:0_22:6	0.037	0.553
TG 14:0_16:1_16:1	0.044	0.487

TG 14:0_16:1_18:0	0.050	0.454
TG 14:0_16:1_18:1	0.066	0.307
TG 14:0_16:1_18:2	0.079	0.216
TG 14:0_18:1_18:1	0.060	0.370
TG 14:0_18:1_18:2	0.087	0.181
TG 14:0_18:2_18:2	0.087	0.170
TG 14:0_20:4_20:4	-0.005	0.930
TG 16:0_16:0_16:0	0.004	0.950
TG 16:0_16:0_16:1	0.079	0.241
TG 16:0_16:0_18:0	-0.122	0.049
TG 16:0_16:0_18:1	-0.032	0.643
TG 16:0_16:0_18:2	0.064	0.359
TG 16:0_16:0_22:6	-0.063	0.315
TG 16:0_16:1_16:1	0.068	0.299
TG 16:0_16:1_18:0	0.043	0.539
TG 16:0_16:1_18:1	0.067	0.321
TG 16:0_16:1_18:2	0.090	0.170
TG 16:0_16:1_20:3	0.010	0.868
TG 16:0_16:1_20:5	0.043	0.497
TG 16:0_16:1_22:5	-0.066	0.285
TG 16:0_16:1_22:6	-0.018	0.773
TG 16:0_18:0_18:0	-0.048	0.433
TG 16:0_18:0_18:1	-0.052	0.404
TG 16:0_18:0_18:2	-0.036	0.560
TG 16:0_18:1_18:1	-0.019	0.765
TG 16:0_18:1_18:2	-0.003	0.961
TG 16:0_18:1_20:1	-0.010	0.865
TG 16:0_18:1_20:4	-0.025	0.689
TG 16:0_18:1_20:5	-0.052	0.416
TG 16:0_18:1_22:6	-0.039	0.537
TG 16:0_18:2_18:2	0.019	0.762
TG 16:0_18:2_20:0	0.040	0.515
TG 16:0_18:2_20:1	-0.023	0.718
TG 16:0_18:2_20:2	-0.027	0.672
TG 16:0_18:2_20:3	-0.015	0.805
TG 16:0_18:2_20:4	-0.042	0.497
TG 16:0_18:2_20:5	-0.022	0.729

TG 16:0_18:2_22:1	0.112	0.075
TG 16:0_18:2_22:5	-0.049	0.432
TG 16:1_16:1_16:1	0.071	0.265
TG 16:1_16:1_18:1	0.082	0.209
TG 16:1_16:1_18:2	0.083	0.192
TG 16:1_16:1_20:4	0.055	0.380
TG 16:1_16:1_20:5	0.039	0.538
TG 16:1_16:1_22:5	-0.032	0.610
TG 16:1_16:1_22:6	0.011	0.855
TG 16:1_18:1_18:1	-0.005	0.937
TG 16:1_18:1_18:2	0.022	0.725
TG 16:1_18:1_20:5	-0.022	0.722
TG 16:1_18:1_22:6	-0.033	0.604
TG 16:1_18:2_18:2	0.055	0.370
TG 16:1_18:2_20:4	-0.024	0.696
TG 16:1_18:2_20:5	-0.001	0.988
TG 16:1_18:2_22:5	-0.050	0.429
TG 16:1_18:2_22:6	-0.044	0.498
TG 18:0_18:0_18:1	-0.044	0.474
TG 18:0_18:1_18:1	-0.042	0.488
TG 18:0_18:1_20:4	-0.043	0.496
TG 18:1_18:1_18:1	0.004	0.943
TG 18:1_18:1_18:2	-0.007	0.909
TG 18:1_18:1_20:4	-0.077	0.214
TG 18:1_18:1_20:5	-0.053	0.404
TG 18:1_18:1_22:5	-0.029	0.641
TG 18:1_18:1_22:6	-0.033	0.599
TG 18:1_18:2_18:2	-0.008	0.901
TG 18:1_18:2_20:2	0.002	0.971
TG 18:1_18:2_20:5	-0.056	0.389
TG 18:1_18:2_22:5	-0.037	0.560
TG 18:1_18:2_22:6	-0.039	0.546
TG 18:2_18:2_18:2	0.002	0.972
TG 18:2_18:2_20:1	0.048	0.455
TG 18:2_18:2_20:5	-0.069	0.289
TG 18:2_18:2_22:5	-0.036	0.575
TG 18:2_20:4_20:4	-0.056	0.375

total TG	0.083	0.207
CE 16:1	-0.068	0.279
CE 18:1	0.027	0.658
CE 18:2	-0.003	0.959
CE 18:3	-0.059	0.341
CE 20:1	0.008	0.899
CE 20:2	-0.009	0.890
CE 20:3	-0.049	0.429
CE 20:4	-0.013	0.832
CE 20:5	-0.016	0.801
CE 22:5	-0.031	0.619
CE 22:6	-0.006	0.920
total CE	-0.013	0.833
Cholesterol	0.034	0.583
PC 14:0_16:0	-0.046	0.463
PC 14:0_18:2	-0.006	0.924
PC 15:0_18:1	0.062	0.333
PC 15:0_18:2	0.047	0.463
PC 15:0_20:3	0.034	0.591
PC 15:0_20:4	0.062	0.354
PC 16:0_16:0	0.043	0.519
PC 16:0_16:1	-0.039	0.545
PC 16:0_17:0	0.073	0.266
PC 16:0_18:0	0.063	0.337
PC 16:0_18:1	0.041	0.539
PC 16:0_18:2	0.072	0.278
PC 16:0_18:3	0.010	0.880
PC 16:0_20:1	0.071	0.268
PC 16:0_20:2	0.030	0.648
PC 16:0_20:3	-0.017	0.809
PC 16:0_20:4	0.014	0.832
PC 16:0_20:5	-0.009	0.884
PC 16:0_22:2	0.079	0.232
PC 16:0_22:4	0.024	0.720
PC 16:0_22:5	0.029	0.644
PC 16:0_22:6	0.070	0.262
PC 16:0e_16:0	0.016	0.812

PC 16:0e_18:1	0.060	0.351
PC 16:0e_18:2	0.056	0.388
PC 16:1_18:0	-0.029	0.655
PC 16:1_18:1	0.036	0.569
PC 16:1_18:2	0.048	0.432
PC 16:1_22:6	-0.001	0.991
PC 17:0_18:1	0.090	0.161
PC 17:0_18:2	0.100	0.114
PC 17:0_20:4	0.033	0.634
PC 17:0_22:6	0.078	0.221
PC 18:0_18:1	0.035	0.592
PC 18:0_18:2	0.062	0.336
PC 18:0_20:2	0.008	0.900
PC 18:0_20:3	-0.021	0.746
PC 18:0_20:4	0.010	0.884
PC 18:0_20:5	-0.027	0.666
PC 18:0_22:5	0.013	0.838
PC 18:0_22:6	0.056	0.370
PC 18:0e_18:1	0.045	0.488
PC 18:0e_18:2	0.064	0.319
PC 18:1_18:1	0.076	0.232
PC 18:1_18:2	0.084	0.182
PC 18:1_18:3	0.013	0.828
PC 18:1_20:1	0.032	0.606
PC 18:1_20:2	0.017	0.790
PC 18:1_20:3	0.012	0.856
PC 18:1_20:4	0.013	0.845
PC 18:1_20:5	-0.014	0.827
PC 18:1_22:5	0.086	0.174
PC 18:1_22:6	0.080	0.199
PC 18:2_18:2	0.058	0.351
PC 18:2_18:3	0.036	0.565
PC 18:2_20:1	0.144	0.027
PC 18:2_20:2	0.026	0.674
PC 18:2_20:3	-0.019	0.770
PC 18:2_20:4	0.055	0.399
PC 18:2_20:5	0.006	0.926

PC 18:2_22:6	0.049	0.452
total PC	0.063	0.330
PE 16:0_18:0	0.157	0.014
PE 16:0_18:1	0.118	0.076
PE 16:0_18:2	0.116	0.075
PE 16:0_18:3	0.064	0.314
PE 16:0_20:3	0.081	0.234
PE 16:0_20:4	0.088	0.190
PE 16:0_20:5	0.068	0.290
PE 16:0p_20:1	0.122	0.060
PE 16:0p_20:2	0.099	0.123
PE 16:0p_20:4	0.040	0.534
PE 16:0p_22:4	0.044	0.500
PE 16:0p_22:5	0.032	0.617
PE 16:0p_22:6	0.036	0.563
PE 17:0_18:2	0.128	0.045
PE 17:0_20:4	0.103	0.117
PE 18:0_18:1	0.120	0.065
PE 18:0_18:2	0.094	0.147
PE 18:0_18:3	0.051	0.426
PE 18:0_20:3	0.065	0.342
PE 18:0_20:4	0.084	0.217
PE 18:0_20:5	0.063	0.318
PE 18:0p_20:4	0.054	0.415
PE 18:0p_20:5	0.069	0.309
PE 18:1_18:1	0.134	0.038
PE 18:1_18:2	0.111	0.082
PE 18:1_18:3	0.079	0.217
PE 18:1_20:3	0.055	0.409
PE 18:1_20:4	0.072	0.286
PE 18:1_20:5	0.056	0.398
PE 18:1p_20:5	0.050	0.444
PE 18:1p_22:5	0.030	0.630
PE 18:1p_22:6	0.032	0.612
PE 18:2_20:4	0.109	0.088
total PE	0.117	0.078
PI 16:0_16:1	-0.046	0.482

PI 16:0_18:0	0.025	0.722
PI 16:0_18:1	0.049	0.460
PI 16:0_18:2	0.012	0.856
PI 16:0_20:2	0.045	0.486
PI 16:0_20:3	-0.003	0.961
PI 16:0_20:4	-0.018	0.781
PI 16:0_22:5	-0.022	0.735
PI 16:0_22:6	0.048	0.477
PI 16:1_18:0	-0.030	0.651
PI 16:1_18:1	0.019	0.761
PI 17:0_18:1	0.092	0.163
PI 17:0_18:2	0.046	0.485
PI 17:0_20:4	-0.005	0.936
PI 18:0_18:0	0.074	0.262
PI 18:0_18:1	0.075	0.254
PI 18:0_18:2	0.033	0.611
PI 18:0_18:3	-0.087	0.162
PI 18:0_20:2	0.067	0.292
PI 18:0_20:3	0.031	0.630
PI 18:0_20:4	0.024	0.713
PI 18:0_20:5	-0.005	0.934
PI 18:0_22:4	0.042	0.532
PI 18:0_22:5	0.032	0.610
PI 18:0_22:6	0.095	0.148
PI 18:1_18:1	0.074	0.253
PI 18:1_18:2	0.045	0.482
PI 18:1_20:3	0.014	0.822
PI 18:1_20:4	-0.014	0.823
PI 19:0_20:4	0.115	0.073
PI 20:0_20:4	-0.002	0.974
total PI	0.032	0.627
PG 16:0_18:1	0.128	0.048
PG 18:0_18:1	0.102	0.132
PG 18:0_18:2	0.093	0.168
total PG	0.120	0.072
PS 18:0_18:1	-0.077	0.217
total PS	-0.077	0.217

LPC 14:0	0.002	0.978
LPC 15:0	0.034	0.583
LPC 16:0	0.066	0.295
LPC 16:1	0.007	0.911
LPC 17:0	0.110	0.089
LPC 17:1	0.063	0.334
LPC 18:0	0.058	0.356
LPC 18:1	0.010	0.875
LPC 18:2	0.010	0.883
LPC 18:3	-0.020	0.752
LPC 19:0	0.129	0.053
LPC 19:1	0.119	0.071
LPC 20:0	0.092	0.177
LPC 20:1	0.095	0.147
LPC 20:2	0.028	0.654
LPC 20:3	-0.039	0.544
LPC 20:4	-0.006	0.924
LPC 20:5	0.034	0.606
LPC 22:0	0.083	0.170
LPC 22:1	0.029	0.658
LPC 22:2	-0.054	0.383
LPC 22:4	-0.056	0.362
LPC 22:5	0.003	0.963
LPC 22:6	0.007	0.909
LPC 23:0	0.029	0.633
LPC 24:0	0.047	0.439
LPC 25:0	0.038	0.528
LPC 26:0	0.040	0.513
LPC 27:0	0.047	0.446
total LPC	0.042	0.508
LPE 16:0	0.024	0.707
LPE 18:0	0.069	0.273
LPE 18:1	0.054	0.399
LPE 18:2	0.005	0.937
LPE 20:4	-0.036	0.565
LPE 22:6	-0.083	0.182
total LPE	0.023	0.715

LPI 18:0	0.055	0.372
LPI 18:1	0.029	0.633
LPI 20:4	0.037	0.558
total LPI	0.038	0.541
Cer d18:1/22:2	0.017	0.809
Cer d18:1/24:0	0.018	0.787
Cer t18:0/20:4	0.004	0.960
Cer t18:0/22:4	0.004	0.958
Cer t18:0/22:5	0.023	0.737
total Cer	0.015	0.827
HexCer d18:1/16:0	0.061	0.365
HexCer d18:1/18:0	0.095	0.131
HexCer d18:1/20:0	0.057	0.374
HexCer d18:1/22:0	-0.014	0.839
HexCer d18:1/23:0	0.053	0.411
HexCer d18:1/24:0	0.013	0.837
HexCer d18:1/24:1	0.092	0.157
total HexCer	0.057	0.394
SM d18:1/12:0	-0.035	0.583
SM d18:1/14:0	-0.014	0.828
SM d18:1/14:1	-0.016	0.812
SM d18:1/15:0	0.021	0.735
SM d18:1/16:0	0.025	0.695
SM d18:1/16:1	0.015	0.812
SM d18:1/17:0	0.051	0.410
SM d18:1/17:1	0.041	0.512
SM d18:1/18:0	0.015	0.818
SM d18:1/18:1	0.027	0.685
SM d18:1/18:2	0.033	0.608
SM d18:1/20:1	-0.013	0.836
SM d18:1/24:0	0.002	0.970
SM d18:1/24:1	0.015	0.817
SM d18:1/25:0	0.072	0.253
total SM	0.016	0.797

Linear regression analysis with adjustment for age, sex, BMI, diabetes duration, hypertension, HbA1c, smoking status (never or ever) and statin use was performed to evaluate the

associations between lipids and carotid intima-media thickness among patients in group 1 (*n*=220).

β, standard partial regression coefficient; FFA, free fatty acid; DG, diacylglycerol; TG, triacylglycerol; CE, cholesterol ester; PC, phosphatidylcholine; PE, phosphatidylethanolamine; PI, phosphatidylinositol; PG, phosphatidylglycerol; PS, phosphatidylserine; LPC, lysophosphatidylcholine; LPE, lysophosphatidylethanolamine; LPI, lysophosphatidylinositol; Cer, ceramide; HexCer, hexosylceramide; SM, sphingomyelin.

Table S2 Internal standards of lipidomic analysis in study 1

Compound name	Additive amount (pmol)
PC 15:0/18:1 (d_7)	2126
PE 15:0/18:1 (d_7)	70
PS 15:0/18:1 (d_7)	64
PG 15:0/18:1 (d_7)	393
PI 15:0/18:1 (d_7)	118
PA 15:0/18:1 (d_7)	102
LPC 18:1 (d_7)	473
LPE 18:1 (d_7)	103
CE 18:1 (d_7)	5322
MG 18:1 (d_7)	55
DG 15:0/18:1 (d_7)	170
TG 15:0/18:1 (d_7)/15:0	678
Cholesterol (d_7)	2542
SM d18:1/18:1 (d_9)	407
Cer d18:1/17:0	50
GlcCer d18:1/12:0	100

PC, phosphatidylcholine; PE, phosphatidylethanolamine; PS, phosphatidylserine; PG, phosphatidylglycerol; PI, phosphatidylinositol; PA, phosphatidic acid; LPC, lysophosphatidylcholine; LPE, lysophosphatidylethanolamine; CE, cholesterol ester; MG, monoacylglycerol; DG, diacylglycerol; TG, triacylglycerol; SM, sphingomyelin; Cer, ceramide; GlcCer, glucosylceramide.

Table S3 Internal standards of lipidomic analysis in study 2

Compound name	Additive amount (pmol)
PC 15:0/18:1 (d_7)	1000
PE 15:0/18:1 (d_7)	70
PS 15:0/18:1 (d_7)	200
PG 15:0/18:1 (d_7)	50
PI 15:0/18:1 (d_7)	200
PA 15:0/18:1 (d_7)	100
LPC 18:1 (d_7)	450
LPE 18:1 (d_7)	20
CE 18:1 (d_7)	2500
FA 16:0 (13C16)	100
MG 18:1 (d_7)	1000
DG 15:0/18:1 (d_7)	150
TG 15:0/18:1 (d_7)/15:0	350
Cholesterol (d_7)	3000
SM d18:1/18:1 (d_9)	200
Cer d18:1 (d_7)/15:0	100
GlcCer d18:1 (d_5)/18:1	100

PC, phosphatidylcholine; PE, phosphatidylethanolamine; PS, phosphatidylserine; PG, phosphatidylglycerol; PI, phosphatidylinositol; PA, phosphatidic acid; LPC, lysophosphatidylcholine; LPE, lysophosphatidylethanolamine; CE, cholesterol ester; FA, fatty acid; MG, monoacylglycerol; DG, diacylglycerol; TG, triacylglycerol; SM, sphingomyelin; Cer, ceramide; GlcCer, glucosylceramide.
